# Gomastahs, Peons, Police and Chowdranies: The Role of Indian Subordinate in the Functioning of the Lock Hospitals and the Indian Contagious Diseases Act, 1805 to 1889

**DOI:** 10.1007/s00048-022-00324-z

**Published:** 2022-02-10

**Authors:** Divya Rama Gopalakrishnan

**Affiliations:** School of Historical and Philosophical Studies, Faculty of Arts, University of Melbourne, Melbourne, Victoria Australia

**Keywords:** Gender, colonial medicine, venereal diseases, Madras, gomastah, lock hospital, caste

## Abstract

Recent scholarship on the social history of health and medicine in colonial India has moved beyond enclavist or hegemonic aspects of imperial medicine and has rather focused on the role of Indian intermediaries and the fractured nature of colonial hegemony. Drawing inspiration from this scholarship, the article highlights the significance of the Indian subordinates in the lock hospital system in the nineteenth century Madras Presidency. This study focuses on a class of Indian subordinates called the “*gomastah*”, who were employed to detect clandestine prostitution in Madras to control the spread of venereal disease. It also underlines the role of other native and non-native subordinates such as Dhais, Chowdranies and Matrons, the ways in which they became indispensable for the smoother operation of the Contagious Diseases Act and the lock hospitals on a day-to-day basis. By emphasising how Indian subordinates were able to bring in caste biases within colonial governmentality, adding another layer to the colonial prejudices and xenophobia against the native population, it underlines the fact that there was not a one-way appropriation or facilitation of the coloniser’s knowledge or biases by the colonised intermediaries. Rather, it argues for an interaction between them, and highlights the complexities of caste hierarchies and prejudice within the everyday colonial governmentality. Moreover, the article focuses on the consequent chaos and inherent power struggle between different factions of colonial staff.

## Abstract (de)

Die jüngere Forschung zur Sozialgeschichte der Gesundheit und Medizin im kolonialen Indien hat sich jenseits spezifischer hegemonialer Aspekte der imperialen Medizin mittlerweile verstärkt der Bedeutung ihrer indischen Vermittler und der gebrochenen Natur kolonialer Herrschaft zugewandt. Davon inspiriert beleuchtet der Beitrag die Rolle indischer Subalterner im System der Krankenhäuser für Geschlechtskrankheiten der Provinz Madras im 19. Jahrhundert. Er fokussiert auf die so genannten „*Gomastah*“, eine Klasse von indischen Untergebenen, die zur Aufdeckung der illegalen Prostitution in Madras eingesetzt wurden, um so die Ausbreitung von Geschlechtskrankheiten zu verhindern. Untersucht wird auch die Rolle anderer einheimischer und nicht einheimischer Untergebener wie der Dhais, Chowdranies und Matrons, die für die reibungslose Umsetzung des *Contagious Diseases Act* und das alltägliche Funktionieren dieser Krankenhäuser unverzichtbar waren. Indem herausgearbeitet wird, wie indisches Kastendenken in die koloniale Gouvernementalität einfließen konnte und so koloniale Vorurteile und Xenophobie stärkte, unterstreicht der Artikel die Tatsache, dass es keine einseitige Aneignung oder Vermittlung kolonialen Wissens und kolonialer Vorannahmen durch die kolonisierten Vermittler gab. Argumentiert wird vielmehr für deren Interaktion, betont wird die komplexe Bedeutung des Kastensystems und seiner Vorurteile für das alltägliche koloniale Regierungshandeln. Zudem befasst sich der Beitrag mit dem daraus resultierenden Chaos und den inhärenten Machtkämpfen zwischen verschiedenen Gruppierungen des kolonialen Personals.

Defendants produced evidence to show that they did protest at the time the Health Office summons was served, and that in fact Thoyee, to whom it was [r]endered, refused to receive it, but that the gomastah threw it down warning them of the consequences.^1^

In 1868, about thirty-seven Indian men were employed by British health officers within the lock hospital system of Madras to detect clandestine prostitution, which they believed was the reason for the increase in venereal disease among British troops. These “detectives” or *gomastahs* became an integral part of the functioning of the Contagious Diseases Act and the lock hospital system in Madras.^2^ Their indispensable position within this system allowed them to regularly employ coercive measures when identifying women who were suspected of being clandestine prostitutes.^3^ The case of Thoyee, which will be discussed further below, clearly displays the plight of women suspected of practising prostitution and the ways in which lower-level staff of the Health Office in Madras used antagonistic and threatening means to compel them to attend the lock hospital medical examination. This article examines the role of the *gomastahs *who worked in the lock hospital system in the Madras presidency. Apart from Sara Hodges (2005) there has not been any significant study on this region in terms of the history of lock hospitals or venereal diseases.^4^ Moreover, it was in Madras where the Indian Contagious Diseases Act of 1868 was first implemented on an experimental basis before it was extended to other parts of India.^5^ While other presidencies relied on police and police peon to identify clandestine prostitution,* gomastahs *were unique to the lock hospital system of Madras presidency.^6^ The article aims to emphasise the significance of Indian intermediaries in the implementation of colonial health policies, such as the Contagious Diseases Acts of 1864 and 1868, and their influence and potential power to manoeuvre it according to their belief and to their advantage. Anchoring the focus of study on the *gomastah* recognises the complex layers of class, caste, and gender in the history of lock hospitals and venereal diseases in colonial Madras. Studying *gomastahs* expands our understanding of colonial bureaucracy, the dominating role of intermediaries, as well as caste prejudices used by them within the lock hospitals. It further contributes to comprehending the gendered notions of caste created by Hindu nationalists in the late nineteenth and early twentieth century.

The historiography of health and medicine in colonial India has traditionally been divided into two sets of literature. The first preoccupation was with the colonial legacy and the question of whether public health facilities flourished under the British government or whether British medicine was enclavist and directed its attention merely to British subjects within the cantonments to the exclusion of Indian subjects (Tinker 1954; Ramasubban 1982; Arnold 1985). A second set of historiographies—owing much to Michel Foucault—saw public health measures under the colonial regime as a means to control its colonial subjects (Arnold 1993; Bynum 1994; MacLeod 1988; Brown 2004). Both approaches looked at the history of health and medicine in colonial India from a simplistic coloniser/colonised binary, ignoring the cracks within the colonial hegemony and in particular the role of Indian intermediaries who helped in the functioning of the colonial project at the ground level.

Recent scholarship, however, has moved beyond the arguments of enclavist or hegemonic aspects of imperial medicine and has focused on different aspects of health and medicine to give a more complete picture of colonial India. Apart from a growing body of literature which analyses the shared medical knowledge and medical markets, which functioned beyond the colonial state’s authority, recent scholarly works have also examined the role of Indian intermediaries, emphasising the fractured nature of colonial hegemony, and highlighting the vital role of Indian agency in policy implementation (Chakrabarti 2014; Bihari Mukerji 2009; Sharma 2009; Guha 2015). None, however, have examined the figure of the *gomastah* in any detail.

Looking at figures like *gomastahs, chowdranies*, and peons means we need to pay more attention to the agency and complexity of Indian subordinate staff during British colonisation. Following Bayly’s lead (1996), recent studies have examined the significant role of Indians agents working within the colonial institutions, especially in medical and healthcare policies. Debjani Das’ work on lunatic asylums in Bengal highlights the ways in which Indian asylum staff became an integral part of the everyday functioning of the institution, so much so that the physicians and surgeons became increasingly dependent on them. As they came to have more control over patients’ day-to-day activities, asylum staff eventually came to replace the outsized figure of the physician in such institutions (2015). The civilian subordinate staff who worked outside the medical circle also played prominent roles in the success of sanitary policies in colonial settings. Amna Khalid’s work is important in this sense, as she discusses the role of colonial subordinate staff who functioned outside the medical institutions or those who were not directly employed in medical infrastructure but made significant contributions to control epidemics and to maintain colonial public health. Khalid has also illustrated how Indian subordinates such as police head constables, constables, and *chowkidars*, though they played a significant role in shaping colonial health measures at the ground level, often also misused their power and exploited the Indian population (2009; Also see Lyons 1994; Bhattacharya et al. 2005; Mills 2000; Saha 2012; Heaton 2013).

Drawing on both the insights and challenges of this scholarship, this article explores the role of Indian subordinates in shaping colonial health measures. The article further interrogates the role of Indian subordinates in exploiting Indian women for their own benefit and investigates the ways in which upper/higher caste subordinate servants working for lock hospitals under the Indian Contagious Diseases Act possibly abused their position in the health office to settle personal scores or class or caste biases. The presence of Indian subordinates working for the lock hospitals and the Indian Contagious Diseases Act, and their misuse of power have been discussed by Kenneth Ballhatchet, Erica Wald and Ashwini Tambe (1980; 2014; 2009). Exploring the role of Indian subordinates in the Madras Presidency broadens and complicates their findings in terms of how the subordinate staff became indispensable for the smoother operation of the Contagious Diseases Act. The article also emphasises how these subordinates inserted caste biases into colonial governmentality, adding another layer to colonial prejudices against the Indian population.^7^

Following Hodges (2005), I emphasise that the subordinate staff implementing the policies at the ground level shaped colonial governmentality. Indian subordinates operated within a colonial bureaucratic order that required them to perform in ways that facilitated the exercise of colonial biopower over colonised patients. However, they found new methods to mould this power in their favour and added their own twist to colonial governmentality. I argue that there was not a one-way appropriation of coloniser’s knowledge, power, or biases by the colonised, but rather an interaction between them, highlighting the complexity of caste hierarchies and prejudices.

This article, using examples from the lock hospital system, emphasises that caste/class-based bias and prejudice against certain communities of women was as much a product of Indian manoeuvring as of the colonial policies of the East India Company and the later British Raj. Although colonialism and colonial officials in nineteenth-century India played an important role to “ethnise” and reify Indian society in terms of caste and religious communities, Indians interacting with colonial officials and working as subordinate staff in colonial institutions also contributed to this reification to some extent. However, it is beyond the scope of this article to explore this in further detail, and indeed it might be impossible to establish the relative role played in ethnising and universalising caste by Indian subordinates due to the lack of sources. Instead, this article aims to show that caste did play an important role in evaluating women who were brought to the lock hospital and demonstrate how caste was mobilised by some of the subordinate staff of lock hospitals to manoeuvre colonial policies at the ground level in their favour. In that sense, caste also became one of the tools of colonial governmentality and surveillance within lock hospitals (Metcalf 1995; Dirks 2001; Fuller 2016; Gupta 2000; Sehrawat 2008; also see, Dutta 2016; Basham 2008; Constable 2001).

## Background

Lock hospitals officially started functioning to control venereal disease among British troops in the cantonments of Cawnpore, Dinapore and Futtugurh from 1797 (Wald 2014:57).^8^ In the Madras Presidency, the English East India company established a system of lock hospitals in 1805 to control the spread of venereal disease among its lower-level employees. The lock hospitals confined prostitutes suspected of being infected with venereal disease (Peers 1998:139). The Royal Commission of 1858, however, suggested that for a stronger control of the “social evil” and better prevention of venereal diseases legislative control was required to give greater power to police and magistrate to detect and confine the “ill-famed” women.^9^ In the wake of the British Contagious Diseases Act of 1864, section 20 of the resulting Cantonment Act of 1864 included clauses for the control and registration of prostitutes. Under this Act, prostitutes were divided into two classes: the first class included public prostitutes frequented by European troops, and the second class included prostitutes not frequented as much by European troops.^10^ Local governments were also allowed to extend the rules to other classes of women living within their jurisdiction in order to control venereal diseases.

The 1864 Act was unsuccessful in curbing venereal diseases among both European soldiers and the prostitutes. Though initially some cantonments recorded a decline, in the following year hospital admissions due to venereal diseases increased considerably.^11^ In 1866, 1868 and 1869, England refined many clauses of the Contagious Diseases Act and extended the number of garrisons and seaports towns under the Act from eleven to eighteen stations. Under this Act, women could be arrested in an area of five to fifteen miles from the station and the regular inspection became obligatory (Hamilton 1978:14).^12^ Following this, the Indian Contagious Diseases Act was passed in 1868. This new Act allowed the local government to make rules and add clauses which would be in accordance with the local environment and circumstances. The word “common” was prefixed to “prostitute” in accordance with revised rules. The Act also brought the brothels and their keepers under inspection and levied heavy penalties, which were not mentioned in earlier Acts, on both parties.^13^ After 1868, local governments conducted the periodical inspection every seven days, instead of every fortnight.^14^

The Act was implemented in the town of Madras on 15 October 1868. It was first applied to Madras in order to test the practical working of the law, before it was extended to other places in the Presidency as well as other parts of the country.^15^ The Act was divided into four parts: prophylactic or preventive, detective, punitive, and sanitation.^16^ It also divided Madras into five districts, each of which had separate health offices that registered and examined women suspected of practising prostitution.^17^ A board was fixed in a prominent place at each of the health offices which explained the procedure of registration in various vernacular languages such as Tamil, Telugu and Hindustani.

Despite these preventive measures, venereal disease continued to increase among the British troops. Surgeons and medical examiners complained about the inefficiency of the police in bringing new or “fresh” batches of women for registration. The continued increase of venereal diseases among the troops led the medical examiners to believe that clandestine prostitution was being practised. This led to the appointment of the *gomastahs* as special detective agents whose duty was to “discover” and report all the clandestine prostitution.^18^

Most Indian women were under suspicion of practising clandestine prostitution. The women who were most targeted however, were the so-called “dishevelled” women. The poor and destitute had always been considered dangerous to society since they were assumed to have the power to corrupt “innocent” gentlemen into their illicit way of life. The lock wards and hospitals often admitted individuals described in hospital reports as “being in many instances, the most destitute of the community; often almost in rags.”^19^ Public prostitutes were not supposed to practice or reside within the cantonment unless they registered and presented themselves for the periodical examination conducted every fortnight by the medical officer. Registered women were given a printed ticket which had to be renewed annually and were not supposed to lend or transfer their ticket to other women.

Women living in or around particular areas, or who were seen in public spaces, were also stigmatized as prostitutes (Ramanna 2000).^20^ Women in urban public spaces, who were often poor, and considered an undesirable section of the population, have been discussed by several scholars (Ryan 1990; Burton 2003; Orsini 2009; Gooptu 2001; Banerjee 1998). The notion of the sharp distinction between private and public led elite Indian men to assume that women in public space were unchaste and the “other”. This maligned image of women in public was used as an example by them to dictate and monitor respectable women’s behaviour and encourage them to remain secluded in their private dwellings (Banerjee 1998). In order to maintain better control over the undesirable section of women in public, the colonial government in Madras gathered women whom they suspected of practicing prostitution into particular areas.^21^ These areas, known as *lal bazzar*, were usually situated near a cantonment but were concealed enough from the respectable community of the city.^22^ Any woman roaming the bazaar or nearby cantonment after a certain time in the evening, or any woman who lived in a certain area or did not have a male relative living with her fell under suspicion of practising prostitution. Such women were issued notice to present themselves in front of the health officer, who would medically examine them. If a woman was found healthy, she was registered and given a registration ticket.^23^ If any woman was found to be affected with any venereal disease, then the woman was sent to the lock hospital and kept under confinement until she was cured. Any woman not registered as a prostitute who was charged with public soliciting was liable for trial before a magistrate.^24^ If a woman was convicted of practising prostitution or soliciting publicly before a magistrate, then she was also liable to be sentenced for imprisonment. The health officer informed the officer in charge of the jail that a specific woman was diseased so that she could undergo medical treatment while being imprisoned. Once she had finished her sentence, she was sent to the health officer for a medical examination, and, if found to be affected with any venereal disease, then she was detained to the lock hospital until cured.^25^ Furthermore, women were also targeted according to their age groups: women below the age of forty were compulsorily examined.^26^ Women roughly between the ages of seventeen to thirty were the most targeted by the medical examiners. Brothel keepers below the age of forty were also subjected to examinations.^27^ All suspected women could be inspected by any police officer, or by the health officer or by any other officer authorised to make such inspection.^28^ This indicates how the system of medical examination provided a window for the subordinate staff to take advantage of the women in public who were suspected of practising prostitution.

## Matrons, Peons, and Police

European policy makers, surgeons, medical examiners, and other European staff appear more prominently in the narrative of the lock hospital system, but these European superiors were heavily reliant on their Indian subordinates for effective performance at the practical level. Indian subordinates had valuable knowledge of the local language, customs, religion as well as the region, which were essential in establishing and maintaining effective colonial control. Imperial masters understood that these Indian subordinates could play a significant role as cultural brokers who could facilitate the process not just of policy implementation and colonial dominance, but also as persons who could also extend colonial surveillance to the ground level.

The lock hospital system—which was believed to be the only way to control the spread of venereal disease and establish surveillance over women considered to be “illicit” and “dangerous”—required the extensive participation of Indian subordinates for its stable and successful operation. Before the *gomastahs* were appointed in the health office of Madras, the matrons,* chowdranies *(Indian brothel keepers), *dhais* (midwives), peons, and police functioned as an essential part of the lock hospital system. In order to understand the pivotal role of *gomastahs*, we need to discuss all the other kinds of subordinates in the system. Discussion of each of these subordinate staff also illuminates the circumstances that led to the appointment of *gomastahs* as the detective force of the Madras lock hospital system.

The matrons in some instances were of European origin: the two lock hospitals in the town of Madras—Vepery and Black Town—each had a European matron.^29^ The matron’s duties were to keep close surveillance over the inmates, to be present during the examination of women in the lock hospitals, and often also to function as a nurse assisting surgeons during treatment.^30^ They frequently also detected and brought diseased women to the lock wards and hospitals.^31^ The matrons were expected to be a beneficial and persuasive influence on the women inside the lock hospitals.^32^ They were supposed to display superior morality and chaste behaviour, which policy makers believed could influence the women under their supervision. The matrons also functioned as disciplinarians and on many occasions caught women who tried to escape the institution.^33^ In 1886, two women Rajah and Lutchumee who tried to escape the lock hospital at Black Town in the early hours of the morning were caught by Mrs O’Toole, the matron of the lock hospital, while they were trying to break out at night and steal hospital clothing. The women appeared at the police court and received a sentence of one month’s rigorous imprisonment.^34^ Although policy makers believed that European matrons were of better influence over the lock hospital inmates and served as better disciplinarians, in practice, matrons of European origin were more expensive to employ and constituted a heavy financial burden on imperial funds.

There was much debate among officials in 1860 on the subject of the appointment of trained European nurses and matrons from England to work in the lock hospitals in Madras.^35^ The discussion not only focused on whether a trained nurse of European origin should look after Indian prostitutes in the lock hospital, but also reviewed the salaries of such trained nurses and matrons which put a financial burden on imperial funds.^36^ The sanitary commissioner J. M. Cunningham concluded that a trained European nurse seemed unnecessary for the lock hospitals in Black Town, where it was presumed that the prostitutes under treatment were all Indians; instead he urged that such trained nurses be reserved for the treatment of the European sick.^37^

Since fewer European matrons and trained nurses were available, and those who were employed were expensive, surgeons often tried to employ both Indian and European brothel keepers. Brothel keepers were often expected to function as matrons of the system of lock hospital within their respective brothels. Such women were also considered well suited for the task since they were familiar with the brothels and the local bazaar. Although brothel keepers had been regularly used since the 1840s to provide information about clandestine prostitution, in 1867 it was suggested by the surgeons that the process of identifying clandestine prostitution would be much easier if brothel keepers could officially form part of the system.^38^ The registered brothel keeper was supposed to maintain a list of all prostitutes, as well as female servants residing in the brothel, and to furnish the list to the health officer and the commissioner of police.^39^ The brothel keepers had to report all cases of disease occurring among the prostitutes of their respective brothels immediately to the health officer.^40^ They were also supposed to force the prostitutes to attend medical examinations once every seven days.^41^

Another set of female subordinate staff employed under the lock hospital system were the *chowdranies* or the *chowdrans* (the Indian equivalent of brothel keepers). They were different from the European and East Indian brothel keepers in the sense that they did not necessarily provide a place of abode to women practicing prostitution.^42^ The *chowdranies* of the bazaar often received a monthly payment from each prostitute in her section as a brokerage fee or *taragu*. This gave the impression that *chowdranies* acted as the servants of the prostitutes. The nature of this relationship caused endless difficulty for the police and other lock hospital servants, as the *chowdranies* had no personal interest in the detection of the disease and often neglected medical inspections, which only hindered their monthly income from the prostitutes.^43^ Under such circumstances, the surgeon J. T. Williams of Kamptee cantonment suggested that the *chowdranies* be given regular payments and bonuses for every diseased prostitute they delivered, while they should be fined for every diseased prostitute who was detected without their cooperation.^44^ Medical examiners believed that the *chowdranies* would comply with the lock system to ensure regular payment and avoid punishment. The *chowdranies* visited the women in their own homes and detected and reported cases of venereal diseases to the police constable and surgeons.^45^

The *dhais* (midwives) were another essential part of clandestine prostitution detection in the late 1870s and mid-1880s in the Madras Presidency. The *chowdrans* often worked with *dhais* to identify diseased women. The two groups often had an indistinguishable role in the lock system: in the 1877 annual lock hospital report of Kamptee, the *chowdran* was referred to as a *dhai*. The *dhais*, apart from performing the duties of the lock system, also had to perform the role of midwifery, which distinguished them from the *chowdrans*. *Dhais’* duties included assisting with the medical examination of suspected women and accompanying the constable of police when he served summons to the prostitutes who failed to attend medical examinations.^46^ They were also expected to visit women suspected of being prostitutes at their homes and examine and report to the surgeon when they were infected.^47^ Of course, the role as a midwife permitted more intimate access to women across class and caste hierarchies. In Madras, this system potentially gave matrons, *dhais* and brothel keepers the opportunity to exploit both prostitutes and women who were under suspicion. The *chowdranies* often abused their position by extracting bribes or other favours from the suspected women. Their greed for a bonus or their fear of being fined or punished led the matrons and *chowdranies* to turn in any woman they suspected of being diseased. Surgeons in-charge of lock hospitals often worried about *chowdranies* abusing their position. In 1849, R. Alexander Lieutenant Colonel of Adjutant General of the Army in Cannanore, lamented that:

Indian establishments of Chowdranees are liable to greatest abuses, and that while diseased women may remain [unnoticeable] to the police, it was by no means an unusual oppression for women in good health, and even poor, respectable females to be threatened with confinement in Lock Hospitals.^48^

Captain Morgan, superintendent of Police at Cannanore, also reported that several people had informed him about the common occurrence of *chowdranies* threatening respectable women with the visit, which would lead to their excommunication and *chowdranies* received donations to keep away.^49^ Such abuses and extortions made the British surgeons discontinue the system of *chowdranies* from 1851 to 1860, but they were reintroduced officially in 1867.^50^ Apart from performing the duties of maintaining surveillance, the *chowdranies* also performed other services. Women suffering from sickness were supposed to report to both *chowdranies* and Peons (lower-level office help), and the *chowdran* of the region supplied them with medicine from the hospital. *Chowdranies* and peons were also appointed to collect two *annas* from dancing girls, cottage women and prostitutes for their service.^51^

A further group of lower-level staff operating in the lock hospital system in Madras presidency were the peons. A peon was a male lower-level staff member whose main function was to exercise surveillance within the institution. They also functioned to prevent inmates from leaving the lock wards and hospitals without the permission of the surgeons and preventing inmates and visitors from smuggling illegal substances, such as tobacco.^52^ The peons often also helped to stop brawling between inmates, checked the pilfering inside the lock hospital and functioned to intimate all necessary information in favour of the lock institution.^53^ In the Bombay presidency the peons also functioned as a detective force and were often selected from the Indian policemen; their duties were to arrest all strange and suspicious women found in or near European regiments (Wald 2014:61; Ballhatchet 1980:16).

The lock system did not just depend on matrons and internal lower-level employees. The system also required an external surveillance staff (such as the police) who had more authoritative power over women under suspicion. The police and police peons were initially employed to detect prostitutes suspected of suffering from venereal disease and to forcefully bring them to the lock hospital for full medical examination and treatment. The police were authorised to arrest any women found publicly soliciting or found in the act of prostitution without a warrant.^54^ The police issued permission to common prostitutes who wished to reside within the limits of any cantonment in Madras.^55^ The commissioner of police also issued permission to brothel keepers who wished to establish a brothel within the limits of the town of Madras. Such brothels had to be registered by the health officer.^56^

The police abused their position by exploiting or extorting bribes from women suspected of practising prostitution. The administrative records often documented the lamentations of senior British officials about abuse by the Indian lower-level staff. These colonial administrators were often critical of subordinate Indian men and women working with the colonial institutions. While working for colonial institutions might have improved these Indian people’s economic and social position, there remained a bias against Indians working as lower-level staff (Dutta 2016; Gupta 2010). Despite their frequent misuse of the position and abuse of the women at their disposal, they formed an integral part of the lock hospital system (Wald 2014:62). However, the lower-level staff were subjected to continuous criticism and their positions were often rotated to keep them in check. The medical superintendent and surgeons constantly complained about the inefficiency of the police in bringing a so-called “fresh” lot of women for inspection. The medical officers often grumbled about police receiving bribes and letting the women who supposedly practise clandestine prostitution off the hook, without entering them in the list of registered prostitutes.^57^ It was suggested by Surgeon N. B. Major of Cannanore that close supervision be established over the police for the purpose of preventing bribery.^58^

## *Gomastahs* and Struggles for Power

It was, then, in the absence of an effective police force that the office of *gomastah* was created. *Gomastahs* were originally men employed as Indian agents for the British East India Company and later for the British Crown, performing everything from clerical jobs to brokerage. The *gomastahs*—part of the police force and known as police *gomastahs*—formed an essential part of the lock hospital systems from as early as 1844.^59^ Under the Indian Contagious Diseases Act of 1868 for Madras, they were employed as a detective agency specifically attached to the Health Office and separate from the ordinary police.^60^ Thirty-five *gomastahs* were appointed in 1868, seven for each division of Madras.^61^ There were six first-class *gomastahs* and between twenty-three and twenty-nine second-class *gomastahs*. There were also some European *gomastahs*. Most of the head *gomastahs *were either of European origin or East-Indians.^62^ Their Indian counterparts belonged to a relatively high caste.^63^ They were specifically selected by the health officer H. Stanbrough and approved by the government committee.^64^ The duties of both Indian and European *gomastahs* were to seek out brothels and other places of resort by prostitutes, to discover and to report all clandestine prostitution, to see that all common prostitutes in their district registered themselves, and to bring before the health officer all cases of unregistered public prostitution.^65^ They were expected to carry out their duties not through coercion or intimidation, but through the gentle persuasion of suspected women to voluntarily submit themselves for lock hospital examination.^66^

*Gomastahs* who worked in lock hospitals present a type of subordinate peculiar to the Madras presidency. This particular type of subordinate distinguishes the lock hospital system in Madras from the other two presidencies. Although on the surface the lock system and the Contagious Diseases Act seemed to have functioned uniformly, the sanitary and surveillance measures were carried out in different ways in all three presidencies to suit their local circumstances. As mentioned earlier, the Contagious Diseases Act of 1868 was first introduced as an experiment in the Madras Presidency and later extended to other presidencies.^67^ The report of the Sanitary Commissioner to the Government of India on the functioning of the Act in all three presidencies also mentioned that different measures were adopted in the different presidency towns to detect or to bring suspected women under surveillance. In Calcutta the police force was the only agency employed to detect clandestine prostitution, whereas in Madras and Bombay the process of detection of clandestine prostitution was mostly handled by a non-police force. In Bombay detectives were assisted by police peons (Ballhatchet 1980:16). The report notes that in Madras the police were expected to check upon a special agency employed to detect clandestine prostitution—most likely referring to *gomastah*.^68^

The term *gomastah* does not appear in any of the lock hospital reports or in the report on the functioning of the Contagious Diseases Act in other presidencies. This particular strand of subordinate class appears only in the earlier lock ward reports of Bellary and Trichinopoly and in the reports on the functioning of the Contagious Diseases Act for the first six months in the Town of Madras.^69^ The term *gomastah* disappears gradually from the annual reports on the lock hospitals and venereal diseases from the mid-1870s onwards. The newspaper reports on the Contagious Diseases Acts and lock hospitals also mention them only until November 1869.^70^ Interestingly, Stanbrough, the Health Officer of Madras in 1869, expected the number of *gomastahs* would be increased by the government to support the Health Office registration after the first six months of the Contagious Diseases Act.^71^ There is no concrete evidence to show why they did not continue to employ *gomastahs* in lock hospitals or why the term disappeared from the records on lock hospitals and control of venereal diseases in Madras. It is possible that they continued to function under some other name. Once the Contagious Disease Act was repealed in 1886, the lock hospitals did not cease to exist but rather continued to function in the name of venereal hospitals until 1903.^72^ Similarly, the colonial officials might have stopped using the term *gomastah,* but the detective force most likely continued to function within the lock hospital system.^73^

The introduction of this new detective force reveals the underlying power struggle between medical officers and the police force. British officials were anxious about abuses of power in general, but this particular aspect highlights the inherent chaos resulting from British officials’ concern for not letting one particular strand of authority gain too much power. This chaotic condition created a space for Indian subordinates (especially *gomastahs*) to misuse their power and to exploit Indian women under suspicion. While the police claimed that until they had enough power to arrest unregistered prostitutes, it would be impossible to impede the spread of the disease, the medical officers wanted less interference from the police and, unless forced by the situation to seek their assistance, they preferred to work with *gomastahs* instead.^74^

Power struggles between the subordinate groups are also evident in a complaint by Surgeon G. B. MacDonell about a police *gomastah* interrupting the duties of the lock ward matron of Trichinopoly Civil Hospital when she escorted two “potentially diseased” “common women” to the hospital. The police *gomastah* took the women from her care at Dark’s Bridge and induced them to return to the Trichinopoly Fort. The surgeon requested the police and police *gomastah* not interfere with the matron in the performance of her duty, and asked that they provide her assistance.^75^

In the 1884 annual medical report on the lock hospital in Bellary, the head police constable complained that an ordinary police constable could not arrest suspected women without a sanction from the superintendent of police or a warrant from a magistrate, which took around ten to fifteen days to be issued. Police constables complained that the fifteen days were enough for the women to hide, abscond or produce fake witnesses.^76^ They also lamented that even when these women were presented in front of a magistrate, the magistrate often allowed them to go without punishment.^77^ The police demanded that they be given the power to bring the women directly to hospital where they would be examined and, if diseased, detained and registered (also Hodges 2005:386).^78^ The medical officers also lamented that the system of police detection of unregistered prostitutes was left too much to the discretion of the magistrate.^79^ They complained that suspected women were often pardoned by the magistrates and that the magistrate continued to insist that the registration of the common prostitutes be voluntary, declining any suggestion of forced prosecution and compulsory registration by the Health Officer.^80^ The Health Officers urged that greater power be given to them in order for the Contagious Diseases Act to function better.^81^

Both police officers and medical officers agreed that the magistrate often did not prosecute suspected women and that they required more power to determine whether or not the woman was a common prostitute. Both groups used their powers to accuse any women they considered to be practising clandestine prostitution. But both groups also found it unpalatable that the other could interfere with their use/misuse of power. The *gomastahs* were appointed during this tug of war between the police officers and health officers. These men thoroughly exploited their power, often using it for their own personal benefit. The fact that health officers preferred them to the police also worked in their favour and allowed them to manipulate the situation for their own benefit. The health officers may have believed that it would be easier to control *gomastahs*, as they were hired on a temporary basis, whereas the police mostly had permanent positions. Moreover, *gomastahs* technically worked under the health officers and did not have a separate office like the police constables did. Of course the *gomastahs* were also suspected of being dishonest and were not completely trusted and there were instances where they were fired for corruption.^82^ But the temporary nature of their employment and their direct subordination to the health officer made them more preferable than the police or police peon.

Health officers were thus most likely left to depend upon the *gomastahs* for information about clandestine prostitution and this enabled the *gomastahs* to have a free reign over the functioning of the process of detection and registration. The *gomastahs* could issue notices on behalf of health officers to women. Upon receiving such notices or summons, the women were required to present themselves to the health office for medical examination and to be registered as common prostitutes. Ignoring such a notice made them liable to punishment. Although the Health Officer H. Stanbrough claimed that such notices could never be issued to any woman without his sanction and stamp, and that the stamp was never available for subordinates or others to make use of, there were instances which show that stamped summons were procured by the *gomastahs* to oppress or exploit the prostitutes and women under suspicion.^83^ Sir Adam Bittleston, lower-level Judge of Madras High Court, in his summary of a case on May 11, 1868 indicated that a *gomastah* has served a summons to a person who was not a registered prostitute, and that the summons had the Health Officer Stanbrough’s name lithographed or stamped upon it. He lamented that this indicated the precarious use of health office stamps and summons by subordinates to oppress any person they wished to.^84^ Moreover, the indispensable nature of their job and overt reliance on and the health officers’ preference for these* gomastahs* over police made it easier for the *gomastahs* to manipulate the situation to their convenience.

## Caste and Governmentality

The subordinate staff employed within the lock hospital and for the Contagious Diseases acts had a coercive position over the prostitutes and women suspected of practising prostitution in the Madras Presidency. The anxiety that accompanied British officials and report writers who relied on subordinate staff about the latter misusing their position to extort from the prostitutes reveals the oppressive power possessed by the Indian subordinates. Surgeons and British officials considered the police, peons, matrons and *gomastahs* absolutely necessary for the successful functioning of the lock hospital system and the maintenance of colonial surveillance, but the reports indicate the strict injunctions that the subordinate staff received to exercise their duties without any use of coercion or intimidation.^85^ The colonial officials often avoided relying on one particular set of subordinates to keep their authority in check. Subordinate staff were also often punished for not reporting the cases properly.^86^ Despite the various attempts by colonial authorities to prevent lower-level Indian staff from exploiting their position, these Indian staff were able to manipulate the system to their advantage.

The Indian subordinate staff, especially Indian men employed in the lock hospital system, did not only have an advantageous position over the prostitutes or women suspected of being prostitutes, but they were also able to influence European surgeons and project their caste biases. This becomes evident in the way that European medical men often used the category of caste to justify their assumptions about Indian society. In 1848, H. R. Milner, Commanding Officer of Ninety Fourth Regiment from Cannanore, wrote a letter to the Major of Brigadier. He mentioned that the venereal disease was increasing daily in the cantonment due to the cantonment being “crowded by diseased prostitutes of the lowest caste” and he further urged the Colonial Government to take some steps to “remedy the evil” before it made the corps under his command inefficient due to widespread disease.^87^ Most of the Indian men, especially *gomastahs*, were employed from a relatively higher caste than the women under suspicion. Most of the women suspected of practising prostitution and being infected by venereal diseases were from the lower class/caste.^88^ It was more often the case health officers registered these women because they were from a lower class/caste.^89^ The subject of British and elite Indian men shaping the sexuality of lower class/caste or Dalit women has been explored by a number of scholars (Banerjee 1998; Gupta 2011, 2016; Burton 2003; Orsini 2009). Charu Gupta highlights how both colonisers and elite Indian men constructed Dalit women as sexually available and loose in moral character, constantly contrasting them with upper caste Hindu women (2011:23). Elsewhere in her recent work, Gupta has also highlighted the ways in which upper caste didactic literature of colonial North India stigmatised Dalit women’s bodies as inherently pollutant, unhygienic and lascivious (2016:28–51). Being lower caste, in some cases, became synonymous with being diseased, and upper caste or “caste” subordinates manoeuvred this classification in their favour.^90^ In that sense, caste prejudices employed by subordinate staff of lock hospitals and especially by *gomastah* were a significant factor in expanding the understanding of gendered caste notions created by Hindu nationalists in the late nineteenth and early twentieth century. These men perceived the mere presence of lower caste/class women in public as something morally dangerous and not in line with their beliefs of an “ideal” woman (Banerjee 1998; Gupta 2011; Burton 2003; Orsini 2009).

Lowest caste women were the most targeted group, and, in some cantonments, these women were specifically examined to keep venereal disease in control. In some instances, surgeons lamented that infections rose at an alarming rate due to the very existence of lower caste women in the cantonment. In 1849, H. R. Milner, the Lieutenant General of the 94th Regiment in Cannanore reported that the overcrowding of lowest caste women in the cantonment has led to an increase in venereal disease among British troops in Cannanore.^91^ Similarly, in a report from 1871, the Secretary of Mysore observed that in the military cantonment only prostitutes or women suspected of being prostitutes of lower class or caste were brought under the Contagious Diseases Act, and the same principle should also be followed in Bangalore where all women were subjected to medical examination under the Contagious Diseases Act.^92^ These examples reveal how caste connotations influenced British officials to the extent that being lower caste and carrying venereal disease became practically synonymous. They also show how the higher caste staff of the lock system were successful in bringing caste prejudice into colonial governmentality, adding another layer of discrimination and prejudice to existing racial stereotypes. These caste connotations often resulted in upper/higher-caste staff misusing power to their benefit. Aside from having power and influence over the lock system, this situation also led them to take financial advantage of women suspected of practising prostitution in the form of bribes to keep away.^93^

Upper/higher-caste subordinate staff were able to influence and bend the rules to their favour. One such case where the *gomastah* misused the summoning notice for his benefit was the case of the health officer of Madras versus Thoyee and Meenatchee in 1869. In this case, these women were judged not just in accordance with their caste or class, but according to where they lived and with whom they lived. These two women lived in an infamous area and lived without a male relative, and these categories were also used against them to prove their offence.^94^ These two women were accused of being common prostitutes duly registered under the Contagious Diseases Act of 1868, but they had neglected to appear for the medical examination by the health officer. The defendants also disputed the validity of their registration and liability under the act. These women were compelled by the *gomastah* to present themselves before the health officer. They claimed:

The Health office goomastah [sic] acting on information imparted by some, who bore them ill-will, procured a notice or summons from the Health Officer, which was served on them. That they then protested and have ever since protested being registered prostitutes, but the goomastah threatened them that if they failed to obey the summons they would be fined twenty five rupees and sentenced to a month’s imprisonment, and told them they [sic] might represent the case to the gentleman (Health Officer). That fearing the consequences of disobeying this summons, they did attend at the Health Office.^95^

The *gomastah* in this case not only served the summons to these women, presuming they were lower-caste common prostitutes based on supposed “information,” but also threatened the women if they refused to oblige. This incident shows how the *gomastahs* might have used their power to score personal vengeance. As in their statement to the high court, Thoyee and Meenatchee claim that they were served the summons because of the information provided by someone who bore them ill-will. Since the source does not reveal much information about the actual reason behind the issue of summons to these two women, I am listing out possible scenarios that led to these two women being targeted by the *gomastah*:

- (i) Thoyee and Meenatchee could have had a quarrel or issue with the *gomastah* in the past, or with someone who was a close ally to the *gomastah*.
- (ii) Another possibility is that the women might have denied the *gomastah* sexual favours.
- (iii) It is also possible that the *gomastah* acted upon the information because the health officer demanded that he detect as many clandestine prostitutes as he could. Hence the *gomastah* might have accused them because the health officer wanted the *gomastah *to bring as many clandestine prostitutes as possible to the Health office before the police intervened.^96^
- (iv) The Health Officer, in his report to Chief Secretary R. S. Ellis, stated that these women lived in *Choolay* with another woman Nagamah, who was supposedly a registered common prostitute: this indicates that Thoyee and Meenatchee were not living with a male relative. The *gomastah* may have assumed that because these women lacked any male family members, they were practising prostitution, or even that they were more susceptible to an accusation without a male relative to defend them.

The actual motive of the *gomastah* is difficult to ascertain, however we can see that his actions were dictated by the fact that these two women lived in *Choolay *and belonged to a lower caste/class.

It is very evident that in the eyes of the *gomastah* they both belonged to a lower caste and were living without a male relative and thus were more likely to be common prostitutes.^97^ Stanbrough questioned the *gomastah* about the women before having any direct interaction with them, and his investigation was already potentially swayed by the *gomastah’s* presentation of the case.^98^ Thoyee and Meenatchee were registered by the health officer because the upper/higher caste *gomastah* had already stated that these women were lower-class common prostitutes.^99^ It is possible that the health officer might have already decided that these women were common prostitutes before they even uttered a word in their defence. According to head *gomastah *Mr. Pope, Thoyee and Meenatchee remained silent when they were asked if they had anything to say in their defence. Their silence was also used against them by the head *gomastah*, who acted as a witness in this case.^100^ Their failure to defend themselves was taken as an affirmation to the questions asked by the surgeon and these women were declared guilty. The investigation seemed like a formality. Although the health officers were suspicious of the conduct of the *gomastahs*, they most often approved their actions and conceded to the caste prejudices that they presented.

Despite the odds stacked against Thoyee and Meenatchee, they were found not guilty on the grounds that the registration was indeed compulsory, as stated in the rules. It should be noted that the person who passed the verdict was the magistrate of police T. Weldon, one of the harshest critics of the health officers serving notices to prostitutes, so much so that he regarded the whole procedure as being illegal. It appears from his report that he favoured the use of police and authorised them to prosecute non-registered women (who were found publicly soliciting). Furthermore, Weldon was of the opinion that the health officer’s power to prosecute should be limited to women duly registered.^101^ He may have favoured the defendants due to the prevailing rivalry between the police forces and the health office, and his biased support to the police. Moreover, it was also noted by R. S. Ellis, Chief Secretary to Government of Madras, that the verdict was in favour of the defendants not because they were respectable women who should not have been served with a notice, “but on the grounds that the health officer had exceeded his powers by the issue of the notice, and on their appearance deciding that they were common prostitutes, and as such, registering their names.”^102^ In practice, however, the government was of the opinion that the issue of notices by the health officer should be continued unless it could be demonstrated that the method was illegal.^103^ Clearly the government wanted to register and confine as many clandestine prostitutes as it could in order to protect its army from venereal diseases. Though the colonial government was quite anxious about the growing power of the health officers, it did not openly oppose the methods used by them or the *gomastahs* to confine women to the lock hospitals.

Another example of a *gomastah* summoning a woman possibly for personal vengeance was the case of the health officer versus Kurreem Bee at the Royapetta Police court in 1869. Bee was summoned for being a prostitute by the health office. The case was dismissed by the magistrate on the grounds that several witnesses denied that Kurreem Bee was a prostitute.^104^ Thoyee, Meenatchee and Kurreem Bee were extremely lucky to have been pardoned by the magistrate. It seems unlikely that other women who were accused of such infamy were as lucky as them. In November 1869, four Indian women were charged at the Black Town police court for having neglected to appear for examination at the health office. Interestingly, one of the women hired a lawyer, Mr. Ward.^105^ Ward successfully claimed that his client was not in Madras on the date when the summons was issued and was simply warned by the *gomastah* on her return to visit the health office for examination, which she promised to do.^106^ Ward pointed out that the Contagious Diseases Act provided a four-day grace period to allow a registered prostitute to appear from the date of her warning. He argued that the time had not elapsed when the *gomastah* summoned her for neglecting to appear for the examination.^107^ Of the four Indian women charged for neglecting the health office notice, the magistrate was convinced that there was no case against the client of Mr. Ward and acquitted her but sentenced three other women to one week’s simple imprisonment.^108^

It was common for a *gomastah* to threaten even a registered prostitute with summons and notices if she refused to comply with his order. Registered prostitutes were even more vulnerable to such exploitation. Mr. Boyd charged an Indian prostitute with refusing to attend the health office examination and refusing to show her ticket. She was summoned to present herself in front of the Black Town police by force and was sentenced to one month’s simple imprisonment and a fine of 50 rupees. Mr. Boyd convinced that the defendant was one whom the provision of the Contagious Diseases Act could touch or one who could be prosecuted without inviting any legal issues from the Magistrate as she herself had registered as a brothel keeper a year before.^109^ Similarly, in March 1871, Thai Ummah, a common prostitute, was charged by the Inspector of Health Office, Mr. R. P. Campbell for failure to attend the Health Office for inspection. The charge being proven, Thai was sentenced to pay a fine of ten rupees after fifteen days of rigorous imprisonment.^110^ This example indicates the miserable position of even the registered prostitutes at the hands of *gomastahs* and health inspectors. This ill-treatment was not limited to registered prostitutes: even dancing girls and kept women were also expected to attend lock hospital health examinations and were often threatened with court summons and imprisonment for failing to do so. On October 19, 1870, Rungaib, a health office *gomastah* of Madras, lodged a complaint in Town Police Court against Rajum, an alleged dancing girl for not attending lock hospital examination. In the course of the court examination Rajum told Mr. Clarke, the Magistrate, that she was a kept woman of Amber Sunker Tauker, and received fifteen rupees from him as monthly allowance, hence was not under the obligation to attend the health office examination. This claim was denied by both Mr. Miller who fought the case on behalf of Tauker, as well as Amber Tauker who denied having any ties to Rajum. He claimed that he knew that she was a dancing girl attached to Ponnery Pagoda but had never met her or had any relationship with her. Upon investigation it was found that Tauker was lying and was indeed in a relationship with Rajum. Having found that his statement was false, Tauker was sentenced to two month’s simple imprisonment by the Magistrate.^111^ Tauker denied his relationship with Rajum and persisted in claiming that she was a dancing girl attached to a pagoda instead of accepting that she was his keep. This example hints at some of the ways in which Indian men used the Contagious Diseases Act to their advantage to exploit a woman, in which the male patrons could easily be left unscathed while the kept women and dancing girls were targeted and scrutinised for being “prostitutes”. However, the coercion and control that the *gomastahs *and lock system attempted to enforce were not absolute: the cases of Kurreem Bee, Thoyee and Meenatchee, Rajum, and Mr. Ward’s unnamed client revealed that these women were able to contest the control through court petitions and sometimes successfully used colonial institutions (in this case, the court) to escape colonial coercion. Sara Hodges (2005) has discussed in detail how during the Madras famine of the mid-1870s, the inmates of lock hospitals reconfigured the institution’s function for their survival. Similarly, examples from the annual lock hospital reports and the reports on the implementation of the Contagious Diseases Act, and in particular the complaint section in these reports, reveal these women’s agency to resist or subvert the hospital space. A number of strategies were available to them: by refusing to attend medical examinations, moving beyond the limits of the Contagious Diseases Act, escaping the lock hospitals, stealing from the lock hospitals, or using the hospital space to avoid starvation, some women contested the coercion and control of the lock hospital system.^112^

## Conclusion

This article has presented the functioning of the lock hospital system in the Madras presidency as a complex interaction of motivations, hierarchies, and processes. Scholars such as Phillipa Levine, Douglas M. Peers, Kenneth Ballhatchet, and Erica Wald have extensively contributed to the study of venereal diseases and lock hospitals in colonial India (1994, 2003; 1998; 1980; 2014). But other than Sara Hodges (2005) and Ashwini Tambe (2009), the works on lock hospitals so far have focused on the significance of race as a category to understand the changing nature of the sexual relationship between the colonisers and colonised and the way lock hospitals functioned as a tool of colonists to control venereal disease and to establish sexual surveillance over British troops and Indian prostitutes. This article highlights the cracks within the colonial policies to control venereal disease and contributes to our understanding of the nature of South Asian historical actors’ “agency” in debates about their role in colonial bureaucracies and the functioning of colonialism.

This article has focused on nuances within these colonial measures and institutions and addresses the fissures within imperial medicine by focussing on the Indian subordinates working in colonial medical institutions such as lock hospitals. In doing so, I show the significant role Indian subordinates played in the functioning of colonial hospitals and other medical infrastructure in Madras. The article also discussed how caste hierarchies shaped the employment and functioning of Indian subordinates in colonial hospitals. The *gomastahs* who played such a significant role in the lock hospital system and implementation of the Contagious Diseases Act of 1868 disappear from the colonial records related to lock hospitals towards the 1870s. The sources do not reveal why this might have happened, although *gomastah* as a clerical post continued to function in other government departments of Madras presidency. It is possible that the police completely took over the process of reporting clandestine prostitution and serving the summons, leaving the Health Office with the responsibility of treatment of the women suspected of having venereal disease. It is also possible that the Health Office of Madras might have changed the term for their detective force, so probably after the 1870s they were known by some other name. It is possible that the police force might have recruited the *gomastah* directly into their forces to identify clandestine prostitution, so they became integrated into the police force. Nevertheless, these Indian upper caste or caste subordinates continued to work within the colonial government and could utilise caste connotations and prejudices to exploit their position within the system to their advantage. Existing literature on intermediaries within colonial institutions has focused on their active participation in the shaping of colonial discourse and agency under colonial governance, but comparatively less attention has been paid to the nature of this agency in terms of caste. The Indian staff became an integral part of the everyday functioning of the lock hospital and the Contagious Diseases Act, so much so that surgeons and medical examiners became increasingly dependent on staff for successful operation at the ground level. Indian subordinates were necessary to facilitate the implementation of surveillance over the “dangers” of clandestine prostitution, which threatened not only the health of British soldiers but also challenged colonial authority more generally. This dependency of colonial authorities on upper/higher caste subordinate staff was clearly exploited and manipulated. Although attempts were made by the colonial authorities to keep these staff in check, Indian subordinates were able to misuse their power and exploit both women practising prostitution and women under suspicion. The lock hospital system synonymised being lower caste or being in public with being diseased. The in-depth study of *gomastah* highlights the presence of a power struggle among Indian subordinates, and the presence of caste components within the lock hospital system in the Madras presidency. Moreover, it expands the historical interpretations of caste as a gendered norm in nineteenth-century India. This process of caste-based characterisation of suspected women within the colonial narrative was to some extent the result of interaction with and influence of upper/higher-caste staff who were able to use caste as a tool to their advantage and brought casteist ideas into the everyday running of lock hospitals.

## Footnotes

1 In Royapet Police Court, Calendar Nos. 247 and 248 of 1869. Health Officer versus Thoyee and Meenatchee, in Report on The Working of the Indian Contagious Diseases Act for the first six months of its introduction written in the Town of Madras. No: 21/23. Home Dept./Public Branch, 18 December 1869.NAI.

2 The term “detective” was used interchangeably in the primary sources by the colonial administrators with the term *gomastah*, who were employed to “detect” clandestine prostitution. They may have functioned more as a substitute police, vigilante or surveillance agents who brought suspected women to lock hospitals. But this was the term used in the records.

3 I use the term “prostitute” instead of “sex worker” because the latter entered the vernacular in the 21st century, and historically the term “prostitute” has been used in my sources. I acknowledge that the term “prostitute” does not sufficiently recognise the agency of present -day sex workers. At the same time using the term “sex worker”, would not only be historically inaccurate but at the same time perpetuate the myth that the term “sex worker” is devoid of the same negative connotations that the term “prostitute” carries. Use of the term “sex work” is still debated as whether it perpetuate the myth that “sex work” means commodified consent to sex, and how such meanings ignore the possible abuse or violence which is associated with this profession. In such a scenario, using the term which my sources also used but with the acknowledgment of the issues and problems associated with the term seemed more feasible (Hunt & Chamberland 2006: 203–16). Similarly, I acknowledge the issues and problems associated with terms such as “common” women, “immoral”, “dishevelled”, “illicit”, “dangerous”, “ill-fame” and “infamy” used throughout this article, and I understand that it might reflect too closely to the language used by the colonial administrators. But for historical accuracy and to show the readers how these women were described in the colonial records with the awareness and caution that these terms in no way reflect the physical, moral, or sexual characters of my historical subjects, I have decided to retain them in my work.

4 The Madras presidency also played a significant role in establishing and maintaining the East India company and the British raj as the colonial overlords of India. Fort St. George or Madras sent reinforcements to capture Calcutta during the Battle of Plassey which transformed the fate of the English East India from mere trading group to overlords of India. Sekhar Bandyopadhyay (2004). Moreover, the British had a stronghold over southern territories longer than the Bengal or Bombay presidency, establishing Fort St. George in as early as 1652.

5 Although the Cantonment Act of 1864 was functioning in other parts of India which had similar features of the Indian Contagious Diseases Act of 1868. From the Hon’ble R. S. Ellis, C. B., Chief Secretary to the Government of Fort St. George to E. C. Bayley, Esquire, Secretary to the Government of India, Simla, -No.698, 9 June 1868 in Home Department Proceedings, Home Dept./Public Branch 10 April, 1869, NAI.

6 The Contagious Diseases Act. Madras Mail (Madras India) 26 September 1872, 2. In the eighteenth and early nineteenth centuries *gomastah* were employed in other presidencies as well, especially there are records of their employment in Bengal presidency where they performed the role of a broker/an agent between weavers and the company (Mitra 1978: 234–237; Reports from Committees of the House of Commons which Have Been Printed by Order of the House: And are Not Inserted in the Journals [1715–1801, Volume 6], Great Britain. Parliament. House of Commons 1806 Great Britain, 226) In the Madras presidency, they were employed in clerical jobs within varied colonial institutions in the nineteenth century (The London Gazette, 21 December 1886, 6453). There is no concrete evidence to prove why colonial officials in Madras presidency preferred *gomastahs* over police and police peon unlike the officials in the other presidencies. But it could be due to the inherent power struggle between different sections of colonial employees. It could also be due to the continued presence of *gomastahs* as employees within colonial institutions of Madras unlike other two presidencies where they functioned outside colonial bureaucracy as brokers or merchants. The meaning of gomastah changed in different presidencies. While in Madras the term *gomastah* was and still is synonymous to a clerical post whereas in the records of Bengal presidency and Bombay presidency they are documented as brokers or merchants who functioned to negotiate between different groups. (Mines 1994; Leavitt 2007; Natarajan 1955; The London Gazette, 21 December 1886, 6453; Reports from Committees of the House of Commons which Have Been Printed by Order of the House: And are Not Inserted in the Journals [1715–1801, Volume 6, Great Britain. Parliament. House of Commons 1806 - Great Britain, 226]).

7 Michel Foucault’s work (1991) on biopower has paved the way for studies on the history of health and medicine in colonial setting in terms of colonial “governmentality” where it was emphasised that health care institutions in colonial settings functioned as new modalities of power where rigid surveillance was established over its subject (Arnold 1993). Sara Hodges (2005) discusses colonial governmentality within lock hospitals of Madras and argues that lock hospitals were constituted as much by the women who were brought to the lock hospitals who were governing their business and survival tactics through the colonial administration of health.

8 Kanpur, Danapur, Fatehgarh of present-day North India.

9 *The Royal Commission, Vol. 2* Appendix Report of the Commissioners Minutes of Evidence. “To Inquire into the Sanitary State of Army in India”; Madras Presidency, 1863, 650.

10 From A. Mackenzie, Esq., Under-Secretary to the Government of Bengal, to the Secretary to the Govt. of India, in the Home Department. Fort William, 10 August 1868. No. 4419 in Proceedings of the Governor General-in-Council, Home Dept./Public Branch, September 1868, NAI.

11 Ibid 58–64. Extracts From Annual Reports of Female or Lock Hospitals For 1867, No-117/120 in Home Department Proceedings, Home Dept./Public Branch, 26 June 1869, NAI.

12 Ibid.

13 Ibid. Papers related to Act XIV of 1868, Home Department, Legislative Branch, Vol. 2, NAI.

14 Ibid. From Major Genl. R. Shubric, Commanding Hyderabad Subsidiary Forces, to The British Resident, Hyderabad in Report on Working of the Lock Hospital at Secunderabad for the year 1869. No. 11, Home Dept./Public Branch, Nos. 41-43, 1870. NAI.

15 Ibid. From the Hon’ble R. S. Ellis, C. B., Chief Secretary to the Government of Fort St. George to E. .C. Bayley, Esquire, Secretary to the Government of India, Simla, No. 698, 9 June 1868 in Home Department Proceedings, Home Dept./Public Branch 10 April 1869, NAI.

16 Ibid. Report on The Working of the Indian Contagious Diseases Act for the first six months of its introduction written in the Town of Madras. No: 21/23. Home Dept./Public Branch, 18 December 1869.NAI. The Contagious Diseases Act, Madras Mail (Madras, India), 26 September 1872, 2

17 Ibid. Vepery, Triplicane including Saint Thomas Mount, Chintadrepettah, Black Town, Royapooram, and these places had the Health offices for the Examination of Prostitutes, the City being divided into five districts the Women belonging to the Jurisprudence of the respective district had to present herself to the respective Health offices. Report on The Working of the Indian Contagious Diseases Act for the first six months of its introduction written in the Town of Madras. No. 21/23. Home Dept./Public Branch, 18 December 1869. NAI.

18 Ibid.

19 Report on Venereal Ward from 1 May 1844 to 30th April 1845, No.151, in Public Consultation Vol.784. TNA.

20 Ibid., See the section on CS Street in Mridula Ramanna, “Control and Resistance”.

21 Erica Wald and Veena Oldenburg have highlighted how the colonial administration’s practice of gathering women in public into one area ignored the nuanced difference between *devdasis, tawaifs *and petty prostitutes by placing them in the same category. The administration’s practice of establishing moral and medical boundaries for women considered prostitutes, criminalised them, and effectively altered their social and legal status. (Wald 2009: 6; Oldenburg 1990).

22 Bazaar means Marketplace or enclosed street where a variety of goods (vegetables to gold) are exchanged and sold. The bazaar also functioned as a place where the brothels could be found and where individual women offered sexual services. (Wald 2014: 61)

23 Report on The Working of the Indian Contagious Diseases Act for the first six months of its introduction written in the Town of Madras. No: 21/23. Home Dept./Public Branch, 18 December 1869.NAI.

24 From the Committee appointed under Contagious Diseases Act, to the Hon’ble R. S. Ellis, C. B, Chief Secretary to the Government of Fort St. George, Dated Madras, 23 July 1868, in Home Dept/Public, Proceedings Nos.93to102, 24 to 26, Index Nos. 13 to 15. 1869. NAI.

25 Ibid.

26 Rules under section 2, 5, 11 and 21 of Act XIV. Of 1868, in Home Department Proceedings, Index Nos.13to15. Home Dept./Public Branch 1869. NAI.

27 Ibid.

28 Ibid.

29 The Contagious Diseases Act, Madras Mail, 26 Sept. 1872, 2

30 *Annual Report for the Military Lock Hospitals of the Madras Presidency for the year 1878*. NSL. http://digital.nls.uk/75107677, 23

31 Report on Venereal Ward from 1 May 1844 to 30 Apr. 1845, No.151, in Public Consultation Vol.784. TNA

32 Report on Venereal Ward from 1 May 1844 to 30 Apr. 1845, No.151, in Public Consultation Vol.784. TNA

33 Breaking Away from the Lock Hospital, Madras Mail (Madras, India), 21 Sept.1886, 5

34 Ibid. The source doesn’t reveal what they mean by “rigorous” imprisonment, but it is assumed to be hard labour.

35 Introduction of trained female nurses into the hospitals of the town of Madras, No. 101-106, Home Dept./Public Branch, 31 October 1860, NAI.

36 Ibid.

37 Ibid.

38 Names of common prostitutes residing in the cantonment Cannanore 17 Nov. 1848, Military Dept./Military, File no. 87 part A, July 1851, NAI.

39 From the Committee appointed under Contagious Diseases Act, to the Hon’ble R. S. Ellis, C. B, Chief Secretary to the Government of Fort St. George, Dated Madras, 23 July 1868, in Home Dept/Public, Proceedings Nos. 93 to 102, 24 to 26, Index Nos. 13 to 15. 1869. NAI.

40 Ibid.

41 Ibid

42 (No.131), No.179, Report on The Working of the Indian Contagious Diseases Act for the first six months of its introduction written in the Town of Madras. No. 21/23. Home Dept./Public Branch, 18 December 1869.NAI.

43 Extracts From Annual Reports of Female or Lock Hospitals For 1867, No.117/120 in Home Department Proceedings, Home Dept./Public Branch, 26 June 1869, NAI.

44 Ibid.

45 *Annual Report for the Military Lock Hospitals of the Madras Presidency for the year 1878*. NSL. http://digital.nls.uk/75107677 , 22.

46 Ibid. 30

47 *Annual Report for the Military Lock Hospitals of the Madras Presidency for the year 1884*.NSL. http://digital.nls.uk/75107677, 12.

48 In reply to letter of the 21st last forward copies of, expel prostitutes & others from cantt & state the cause of delay, Military Dept./Military, File No: 87 part C, July 1851, NAI.

49 From Brigadier J.P. James Commanding Hyderabad Subsidiary force to The Adjutant General of the Army Fort St. George, Military Dept./Military, File No.118, July 1851, NAI.

50 Extracts from Annual Reports of Female or Lock Hospitals For 1867, No.117/120 in Home Department Proceedings, Home Dept./Public Branch, 26 June 1869, NAI.

51 From Brigadier J.P. James Commanding Hyderabad Subsidiary force to The Adjutant General of the Army Fort St. George, Military Dept./Military, No 973, July 1851, NAI.

52 Conveying Tobacco into the Lock Hospital, Madras Mail (Madras, India), 23 June1875, 2.

53 Report on Venereal Ward from 1 May 1844 to 30 April 1845, No.151, in Public Consultation Vol. 784. TNA.

54 Memorandum on Act XIV of 1868, in Report on The Working of the Indian Contagious Diseases Act for the first six months of its introduction written in the Town of Madras. No: 21/23. Home Dept./Public Branch, 18 December 1869. NAI

55 No. 58 Report on The Working of the Indian Contagious Diseases Act for the first six months of its introduction written in the Town of Madras. No: 21/23. Home Dept./Public Branch, 18 December 1869.NAI

56 From the Committee appointed under Contagious Diseases Act, to the Hon’ble R. S. Ellis, C. B, Chief Secretary to the Government of Fort St. George, Dated Madras, 23 July 1868, in Home Dept/Public, Proceedings Nos.93to102, 24 to 26, Index Nos. 13 to 15. 1869. NAI.

57 *Annual Report for the Military Lock Hospitals of the Madras Presidency for the year 1887*. NSL. http://digital.nls.uk/75107677, 11.

58 Ibid.

59 Report on Venereal Ward from 1 May 1844 to 30 April 1845, No.151, in Public Consultation Vol.784. TNA.

60 From the Committee appointed under Contagious Diseases Act, to the Hon’ble R. S. Ellis, C. B, Chief Secretary to the Government of Fort St. George, Dated Madras, 23 July 1868, in Home Dept/Public, Proceedings Nos.93to102, 24 to 26, Index Nos. 13 to 15. 1869. NAI.

61 Ibid. The town of Madras was divided into five divisions of Health offices 1. Triplicane, 2. Chintadrepettah, 3. Vepery, 4. Black Town, 5. Royapooram.

62 The Contagious Disease Act, *Madras Mail* (Madras, India), October 6, 1869, Digitised by World Newspaper Archives, 4.

63 The Indian *gomastahs *did not always come from upper caste, though they were relatively higher in caste hierarchy than the women who were suspected of practicing prostitution. However, it was highly likely that the Indians employed as *gomastahs* belonged to a better socio-economic condition since in order to be employed in a health office they needed to produce a good recommendation from “respectable persons”. Given how strong the caste hierarchies and networks were in nineteenth-century Madras it was highly likely that these men belonged to a social group which had relatively better status in the caste hierarchy and were able to use their social ties to be employed in colonial institutions. (No.131), No.179, Report on The Working of the Indian Contagious Diseases Act for the first six months of its introduction written in the Town of Madras. No. 21/23. Home Dept./Public Branch, 18 December 1869. NAI.

64 Ibid.

65 Ibid.

66 From the Committee appointed under Contagious Diseases Act, to the Hon’ble R. S.Ellis, C. B, Chief Secretary to the Government of Fort St.George, Dated Madras, the 23 July 1868, in Home Dept/Public, Proceedings Nos.93to102, 24 to 26, Index Nos. 13 to 15. 1869. NAI.

67 The Contagious Diseases Act. Madras Mail (Madras India) 26 September 1872, 2.

68 The Contagious Diseases Act. Madras Mail (Madras India) 26 September 1872, 2.

69 Report on The Working of the Indian Contagious Diseases Act for the first six months of its introduction written in the Town of Madras. No: 21/23. Home Dept./Public Branch, 18 December 1869. NAI. Report on Venereal Ward from 1 May 1844 to 30 April 1845, No.151, in Public Consultation Vol.784. TNA.

70 Madras Mail (Madras India) 1868-1900.

71 (NO.131), NO.179, Report on The Working of the Indian Contagious Diseases Act for the first six months of its introduction written in the Town of Madras. No: 21/23. Home Dept./Public Branch, 18 December 1869.NAI.

72 Question of rendering the law in force in Madras and Calcutta as regards prostitution, March 1903, in Home Dept/Police‑A, Proceedings Nos.79-89. 1903. NAI.

73 Annual report on the Military Lock Hospital of the Madras Presidency for the year 1877. Published by Authority Madras: Printed by R. Hill, at the Government Press. 1878. NSL. http://digital.nls.uk/75107677_31_60, 23-24.

74 Ibid. Annual report on the Military Lock Hospital of the Madras Presidency for the year 1884. Published by Authority Madras: Printed by E. Keys, at the Government Press. 1885. NSL. http://digital.nls.uk/75107677. (No.136), No. 58, Report on The Working of the Indian Contagious Diseases Act for the first six months of its introduction written in the Town of Madras. No. 21/23. Home Dept./Public Branch, 18 December 1869. NAI.

75 Report on Venereal Ward from 1 May 1844 to 30 April 1845, No. 151, in Public Consultation Vol.784. TNA.

76 Annual report on the Military Lock Hospital of the Madras Presidency for the year 1884. Published by Authority Madras: Printed by E. Keys, at the Government Press. 1885. NSL. http://digital.nls.uk/75107677. 10

77 Ibid.

78 Ibid.

79 Ibid., Extracts from Annual Reports of Female or Lock Hospitals For 1867, No. 117/120 in Home Department Proceedings, Home Dept./Public Branch, 26 June 1869. NAI.

80 Ibid.

81 Ibid.

82 (No.131), No.179, Report on The Working of the Indian Contagious Diseases Act for the first six months of its introduction written in the Town of Madras. No. 21/23. Home Dept./Public Branch, 18 December 1869. NAI. The British attitude towards subordinate employees, and episodes of subordinate staff’s corruption and misconduct has been studied extensively in the case of British Burma. See also Saha (2012, 2013); Takahiro (2018).

83 (No.135), Ibid.

84 Ibid

85 Ibid.

86 From Brigadier J. P. James Commanding Hyderabad Subsidiary force to The Adjutant General of the Army Fort St. George, Military Dept./Military, No 973, July 1851, NAI.

87 In reply to letter of the 21st last forward copies of, expel prostitutes & others from cantt & state the cause of delay, No. 1102, Military Dept./Military, File No: 87-part C, July 1851, NAI.

88 The Contagious Diseases Act. Madras Mail (Madras India) 26 September 1872, 2.

89 In Royapet Police Court, Calendar Nos. 247 and 248 of 1869. Health Officer versus Thoyee and Meenatchee, in Report on The Working of the Indian Contagious Diseases Act for the first six months of its introduction written in the Town of Madras. No: 21/23. Home Dept./Public Branch, 18th December 1869.NAI

90 In their work on leprosy in colonial Orissa, Biswamoy Pati and Chandi Nanda have also emphasised how colonialists often assumed and commented that the lower class and its habits were the source of infection which caused leprosy in Puri. (Pati &.Nanda 2009:115). By “caste” subordinates I mean those who did not necessarily belong to the highest in the caste hierarchy but were within its system or rather the lower caste who were not “untouchables”. For more, see B. R Ambedkar, *Annihilation of Caste*: *The Annotated and Critical Edition*, (New Delhi: Navayana, 2016). Also, the “caste” and lower caste men towards the end of the nineteenth century demonstrated their masculinity in aggressive forms by imitating and reflecting the upper caste men’s ideas of chastity, nationalism and communality etc. (Gupta 2010:330-332)

91 In reply to letter of the 21st last forward copies of , Expel prostitutes & others from cantt & state the cause of delay, Military Dept./Military, File No: 87 part C, July 1851, NAI.

92 Suicide committed by an Indian female in Mysore to avoid periodical examination under Lock Hospital Act. Home Dept./Police, File no. 65, October 1872, NAI.

93 *Annual Report for the Military Lock Hospitals of the Madras Presidency for the year 1887*. NSL. http://digital.nls.uk/75107677 , 11. In reply to letter of the 21st last forward copies of expel prostitutes & others from cantt & state the cause of delay, Military Dept./Military, File No: 87-part C, July 1851, NAI.

94 In Royapet Police Court, Calendar Nos. 247 and 248 of 1869. Health Officer versus Thoyee and Meenatchee, in Report on The Working of the Indian Contagious Diseases Act for the first six months of its introduction written in the Town of Madras. No: 21/23. Home Dept./Public Branch, 18 December 1869. NAI

95 Ibid.

96 Ibid. (No.136), Report on The Working of the Indian Contagious Diseases Act for the first six months of its introduction written in the Town of Madras. No: 21/23. Home Dept./Public Branch, 18 December 1869. NAI.

97 Ibid.

98 In Royapet Police Court, Calendar Nos. 247 and 248 of 1869. Health Officer versus Thoyee and Meenatchee, in Report on The Working of the Indian Contagious Diseases Act for the first six months of its introduction written in the Town of Madras. No: 21/23. Home Dept./Public Branch, 18 December 1869. NAI.

99 Ibid. (NO.136), Report on The Working of the Indian Contagious Diseases Act for the first six months of its introduction written in the Town of Madras. No: 21/23. Home Dept./Public Branch, 18 December 1869. NAI.

100 Ibid.

101 Ibid. Memorandum on Act XIV of 1868, in Report on The Working of the Indian Contagious Diseases Act for the first six months of its introduction written in the Town of Madras. No: 21/23. Home Dept./Public Branch, 18 December 1869. NAI

102 Ibid. Order Thereon No.1162, 19 August 1869, by R. S. Ellis, in Report on The Working of the Indian Contagious Diseases Act for the first six months of its introduction written in the Town of Madras. No: 21/23. Home Dept./Public Branch, 18 December 1869. NAI.

103 Ibid.

104 The Indian Contagious Diseases Act, *Madras Mail *(Madras, India), 9 June 1869, Digitised by World Newspaper Archive, 3.

105 Prosecution Under the Indian Contagious Diseases Act, *Madras Mail* (Madras, India), 11 November 1869, Digitised by World Newspaper Archives, 3.

106 Ibid.

107 Ibid.

108 Ibid. It is not evident from the source what “simple” imprisonment means, but it is assumed that it did not involve hard labour.

109 The Contagious Diseases Act, Madras Mail (Madras, India), 2 October 1869, 4.

110 Health Office Prosecution, *Madras Mail *(Madras, India), 27 March 1871, Digitised by World Newspaper Archive, 2.

111 Perjury, *Madras Mail *(Madras, India), 3 November 1870, Digitised by World Newspaper Archive 4.

112 Breaking Away from the Lock Hospital, Madras Mail (Madras, India), 21 September 1886. 5. Extracts From Annual Reports of Female or Lock Hospitals For 1867, No-117/120 in Home Department Proceedings, Home Dept./Public Branch, 26 June 1869, NAI. (No.131) & (No.136), Report on The Working of the Indian Contagious Diseases Act for the first six months of its introduction written in the Town of Madras. No. 21/23. Home Dept./Public Branch, 18 December 1869. NAI.—From Colonel R. D. Ardagh, Commissioner of Pegu Division to The Secretary to the Chief Commissioner of British Burma, No.383–91, Home Dept./Sanitary, May 1876. NAI.—Annual Report on The Lock Hospital of The Madras Presidency for the year 1877, Published by Authority, Madras, Printed by R. Hill, At the Government Press 1878. NLS. http://digital.nls.uk/75107677. 4

## References

[CR34] - (2003). Prostitution, Race and Politics Policing Venereal Disease in the British Empire.

[CR25] - (2010). Feminine, Criminal or Manly?: Imaging Dalit Masculinities in Colonial North India. The Indian Economic & Social History Review.

[CR50] - (2013). Law, Disorder and the Colonial State: Corruption in Burma c.1900.

[CR1] Arnold D (1985). Medical priorities and practice in nineteenth-century British India. South Asia Research.

[CR2] Arnold D (1993). Colonizing the Body: State Medicine and Epidemic Disease in Nineteenth Century India.

[CR4] Ballhatchet K (1980). Race, Sex and Class under the Raj: Imperial Attitudes and Policies and their Critics, 1793–1905.

[CR5] Bandyopādhyāẏa Ś (2004). From Plassey to partition: a history of modern India.

[CR6] Banerjee S (1998). Under the Raj: Prostitution in Colonial Bengal.

[CR7] Basham A (2008). Untouchable Soldiers: The Mahars and the Mazhbis.

[CR9] Bayly C (1996). Empire and Information: Intelligence Gathering and Social Communication in India, 1780–1870.

[CR10] Bhattacharya S, Harrison M, Worboys M (2005). Fractured States: Smallpox, Public Health and Vaccination Policy in British India, 1800–1947.

[CR11] Brown SH (2004). A Tool of Empire: The British Medical Establishment in Lagos, 1861–1905. The International Journal of African Historical Studies.

[CR12] Burton A (2003). Dwelling in Archive.

[CR13] Bynum WF (1994). Science and practice of medicine in the nineteenth century.

[CR14] Chakrabarti P (2014). Medicine and Empire: 1600–1960.

[CR15] Constable P (2001). The Marginalization of a Dalit Martial Race in Late Nineteenth- and Early Twentieth-Century Western India. The Journal of Asian Studies.

[CR16] Das D (2015). Houses of Madness: Insanity and Asylums of Bengal in Nineteenth Century India.

[CR17] Dirks N (2001). Castes of Mind: Colonialism and making of Modern India.

[CR18] Dutta M (2016). The Army as a Tool for Social Uplift: The Experience of the Paraiyans in the Madras Presidency Army, 1770–1895. Social Scientist.

[CR19] Foucault M, Burchell G (1991). Governmentality. The Foucault Effect: Studies in Governmentality.

[CR20] Fuller C (2016). Colonial Anthropology and the Decline of the Raj: Caste, Religion and Political Change in India in the Early Twentieth Century. Journal of the Royal Asiatic Society.

[CR21] Gooptu N (2001). The Politics of the Urban Poor in Early Twentieth-Century India.

[CR22] Guha R (2015). Native Bodies, Medical Market and “Conflicting” Medical “Systems”: Venereal Diseases and the “Vernacularization” of Western Medical Knowledge in Colonial Bengal. Presidency Historical Review.

[CR61] Gupta C (2010). Feminine, Criminal or Manly?: Imaging Dalit Masculinities in Colonial North India. The Indian Economic & Social History Review.

[CR23] Gupta C (2011). Writing sex and sexuality: Archives of colonial North India. Journal of Women’s History.

[CR24] Gupta C (2016). The Gender of Caste: Representing Dalit in print.

[CR26] Gupta D (2000). Interrogating Caste: Understanding Hierarchy and Difference in Indian Society.

[CR59] Hamilton, Margaret 1978. Opposition to the Contagious Diseases Act, 1864–1886. *Albion: A Quarterly Journal Concerned with British Studies,* (10:1). 11614153

[CR57] Heaton MM (2013). Black skin, white coats: Nigerian psychiatrists, decolonization, and the globalization of psychiatry, New African histories.

[CR28] Hodges SD (2005). “Looting” the Lock Hospital in Colonial Madras during the famine years of the 1870s. Social History of Medicine.

[CR29] Hunt G, Chamberland L (2006). Is Sex Work? Re-Assessing Feminist Debates about Sex, Work, and Money. Labour/Le Travail.

[CR30] Iwaki Takahiro 岩城高広 2018. Burmese Subordinate Officials and British Colonial Rule in the Late Nineteenth Century: Significance of Petitions Submitted by a Circle Headman of the Thongwa District. *Journal of Asian History* (52:2): 287–309.

[CR32] Khalid A, Pati B, Harrison M (2009). “Subordinate” negotiations: Indigenous staff, the colonial state and public health. The social history of health and medicine in colonial India.

[CR35] Leavitt D (2007). The Indian Clerk.

[CR33] Levine P (1994). “Venereal Disease, Prostitution and the Politics of Empire”: The Case of British India. Journal of History of Sexuality.

[CR36] Lyons M, Engels D, Marks S (1994). The Power to Heal: African Auxiliaries in Colonial Belgian Congo and Uganda. Contesting Colonial Hegemony: State and Society in Africa and India.

[CR37] MacLeod R, MacLeod R, Lewis M (1988). Preface. Disease, Medicine, and Empire: Perspectives on Western Medicine and the Experience of European Expansion.

[CR58] Metcalf T (1995). Ideologies of the Raj.

[CR38] Mills JH (2000). Madness, Cannabis and Colonialism: The “Native-Only” Lunatic Asylums of British India, 1857–1900.

[CR39] Mines M (1994). Public Faces, Private Lives: Community and Individuality in South India.

[CR40] Mitra DB (1978). The cotton weavers of Bengal, 1757–1833.

[CR41] Mukharji PB, Biswamoy P, Harrison M (2009). Pharmacology, Indigenous Knowledge, Nationalism: Few Words from the Epitaph of Subaltern Science. Social History of Health and Medicine in Colonial India.

[CR42] Natrajan J (1955). History of Indian Journalism.

[CR43] Oldenburg V (1990). Lifestyle as Resistance: The Case of the Courtesans of Lucknow, India. Feminist Studies.

[CR44] Orsini F (2009). Print and Pleasure: Popular literature and entertaining fictions in colonial north India.

[CR45] Pati B, Nanda CP, Pati B, Harrison M (2009). The leprosy patient and society: Colonial Orissa 1870s–1940s. The Social History of Health and Medicine in Colonial India.

[CR46] Peers DM (1998). Soldiers, Surgeons, and the Campaign to Combat Sexually Transmitted Diseases in Colonial India. Medical History.

[CR60] Ramanna M (2000). Control and Resistance: The Working of the Contagious Diseases Acts in Bombay City. Economic and Political Weekly.

[CR47] Ramasubban R (1982). Public health and medical research in India: their origins under the impact of British colonial policy.

[CR48] Ryan MP (1990). Women in Public: Between Banners and Ballots, 1825–1880.

[CR49] Saha J (2012). “Uncivilized Practitioners”: Medical Subordinates, Medico-Legal Evidence and Misconduct in Colonial Burma, 1875–1907. South East Asia Research.

[CR51] Sehrawat S, Pati B, Harrison M (2008). Prejudices clung to by the natives: Ethnicity in the Indian army and hospitals for sepoys, c.1870s–90s. The Social History of Health and Medicine in Colonial India.

[CR52] Sharma M, Pati B, Harrison M (2009). Creating a Medical Consumer: An Analytical Study of Advertisements. Social History of Health and Medicine in Colonial India.

[CR53] Tambe A (2009). Codes of Misconduct: Regulating Prostitution in Late Colonial Bombay.

[CR54] Tinker HR (1954). The Foundations of Local Self-Government in India, Pakistan and Burma.

[CR55] Wald E (2009). From Begums to Bibis to Abandoned Females and Idle Women: Sexual Relationships, Venereal Disease and the Redefinition of Prostitution in Early Nineteenth-Century India. Indian Economic Social History Review.

[CR56] Wald E (2014). Vice in the Barracks: Medicine, the military and the making of colonial India, 1780–1868.

[CR3] Arnold D, Ernst W, Harris B (1999). An ancient race outworn”: malaria and race in colonial India, 1860–1930. Race, Science and Medicine, 1700–1960.

[CR31] Kapila S (2007). Race Matters: Orientalism and Religion, India and beyond c. 1770–1880. Modern Asian Studies.

